# Giant Cell Tumor of the Temporal Bone with Direct Invasion into the Middle Ear and Skull Base: A Case Report

**DOI:** 10.1155/2012/690148

**Published:** 2012-04-03

**Authors:** Takashi Iizuka, Masayuki Furukawa, Hisato Ishii, Misato Kasai, Chieri Hayashi, Hajime Arai, Katsuhisa Ikeda

**Affiliations:** ^1^Department of Otorhinolaryngology, Juntendo University Faculty of Medicine, 2-1-1 Hongo, Bunkyo-ku, Tokyo 113-8421, Japan; ^2^Department of Neurosurgery, Juntendo University Faculty of Medicine, 2-1-1 Hongo, Bunkyo-ku, Tokyo 113-8421, Japan

## Abstract

Giant cell tumor (GCT) is classified as a benign bone tumor, and it is frequently identified at the epiphysis of long bones and relatively rare in the temporal bone. For orthopedists expert at recognizing bone and soft tissue tumors, the diagnosis of GCT is relatively easy; however, since head and neck surgeons experience few cases of GCT, it may be difficult to diagnose when it occurs in the temporal bone. A 32-year-old man complained of left hearing loss, aural fullness, and tinnitus. Examination of the ear revealed a bulging tumor. Audiologic examination demonstrated conductive hearing loss of the left ear. Computer tomograph of the temporal bone showed a soft-tissue-density specification indicating bone destruction at the left temporal bone. The tumor invaded the skull base. Imaging examinations using magnetic resonance imaging revealed a nonhomogenous isosignal intensity area on T1 at the left temporal bone. After intravenous gadolinium, the mass showed unequal enhancement. This patient subsequently underwent surgery to remove the lesion using transmastoid and middle fossa approach. Pathological examinations from specimens of the tumor revealed characteristic of GCT. No clinical or radiological evidence of tumor recurrence was detected for 4 years.

## 1. Introduction

 Giant cell tumor (GCT) is classified as a benign bone tumor, and it represents 4–9.5% of all bone tumors and 20% of all benign bone tumors [1]. It occurs in 20–45-year-old individuals with a slightly higher predominance in woman [2]. The tumor is frequently identified at the distal femur, and proximal tibia, distal radius, proximal humerus and sacrum [3]. Its occurrence is relatively rare in the temporal bone. We report one patient who suffered from an extensive GCT of the temporal bone with invasion into the contiguous tympanic cavity, external ear canal and skull base.

## 2. Case Presentation

 A 32-year-old man complained of left aural fullness in August 2005. He had no past medical or family history. In addition, he experienced left hearing loss and left tinnitus in July 2007. He consulted an ENT practitioner, but his symptoms were not improved. Thereafter, left temporal bone tumor was revealed in a computed tomography (CT) scan. There was no history of headache, nausea, vomiting, or other neurological symptoms. He was referred to our hospital for further evaluation and management in September 2007.

**Figure 1:**
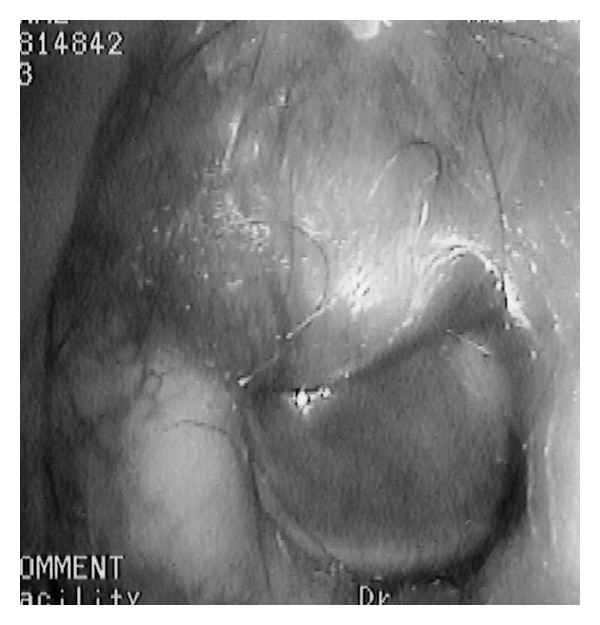
Otoscopy of the left ear. The subcutaneous, bulging tumor was seen from the flaccid part to the umbo of the left tympanic membrane.

Examination of the ear revealed a bulging, subcutaneous bulging tumor from the flaccid part to the umbo of the left tympanic membrane (Figure 1). Audiologic examination demonstrated conductive hearing loss of the right ear with an air-bone gap. The cranial nerve examination showed that the cochlear nerve was intact.

**Figure 2:**
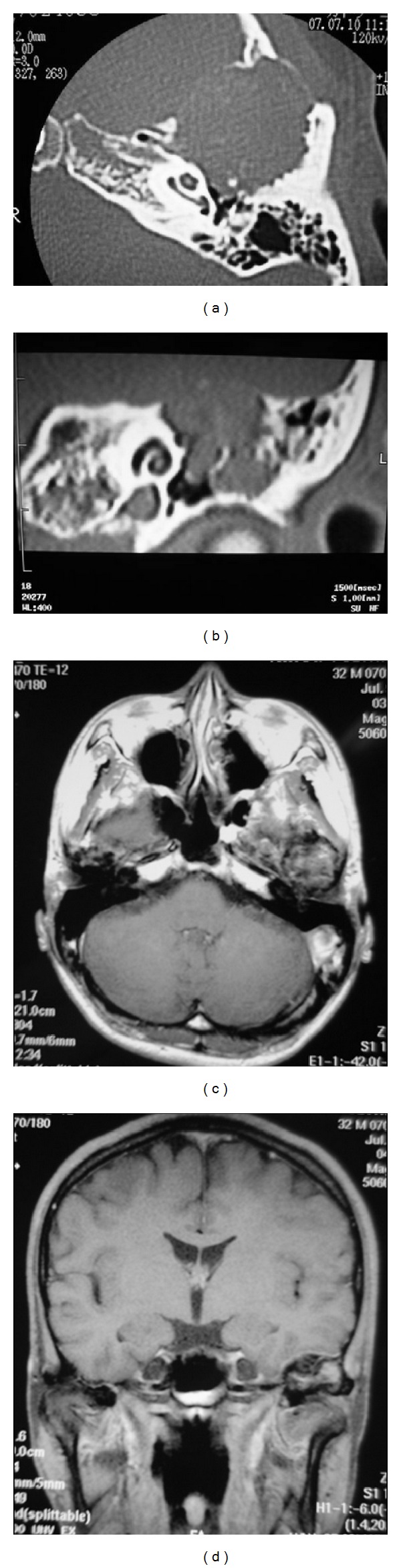
Temporal bone computed tomographic scan shows soft tissue shadow from the bone destruction with massive extension to the epitympanum and ossicular chain (a). In the coronal section, the tumor invades the skull base, and the outline of the tumor shows osteosclerosis with decalcification (b). Magnetic resonance imaging of the head revealed a mass 3 cm in diameter at the left temporal bone that was unequally enhanced after intravenous gadolinium (c). At the coronal section, the border with the tumor and the left mandible head was indistinct. In addition, the dura mater of the middle cranial fossa showed enhancement (d).

CT of the temporal bone showed a soft-tissue-density specification indicating bone destruction, 3 cm in size, at the left temporal bone with massive extension to the mastoid antrum and the temporomandibular joint (Figure 2(a)). Coarse calcification was seen inside the tumor. The tumor extended to the anterior wall of the left external auditory canal, resulting in narrowing of the canal. Furthermore, the tumor reached the facial nerve and the middle ear, and ossicles were embedded in the tumor. In the coronal section, the tumor invaded the skull base, and the outline of the tumor showed osteosclerosis with decalcification (Figure 2(b)).

Imaging examinations using magnetic resonance imaging (MRI) revealed a low-signal-intensity area on T2-weighted images and a nonhomogenous high-signal-intensity area on T1-weighted images that measured 3 cm in diameter at the left temporal bone. After intravenous gadolinium, the mass showed unequal enhancement (Figure 2(c)). In the coronal section, the border between the tumor and the left mandible head was indistinct. In addition, the dura mater of the middle cranial fossa appeared to be involved by the tumor based on the dural enhancement (Figure 2(d)).

**Figure 3:**
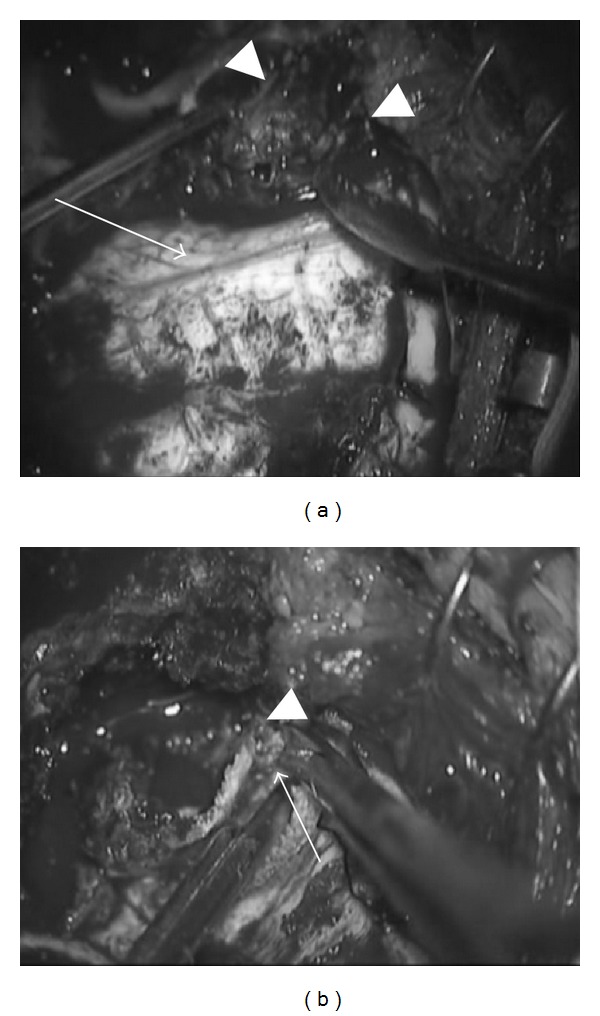
Intraoperative photographs. Transmastoid and middle fossa approach. The tumor was peeled from the middle fossa dura. Arrowhead: giant cell tumor, arrow: dura of the middle cranial fossa.

The patient subsequently underwent surgery to remove the lesion. We confirmed that the tumor did not reach the stapes, and therefore the incudostapedial joint was detached. After mastoidectomy, a part of the tumor around the incus was examined by frozen section. We removed the tumor using transmastoid and middle fossa approach (Figure 3). The diagnosis of the frozen section during the operation was GCT. We peeled the tumor from the middle fossa dura. We removed the incus and the head of the malleus and confirmed the widely exposed horizontal portion of the facial nerve. The entire tumor was then extirpated. Ossiculoplasty was performed with the columella on the stapes. After the operation, the patient experienced mild facial palsy of the left side, but the facial palsy had almost completely recovered by 12 months after the surgery. Postoperatively, otoscopic examination became normal. The pure tone audiometer showed that his hearing improved to a normal level. No clinical or radiological evidence of tumor recurrence was detected for 4 years.

**Figure 4:**
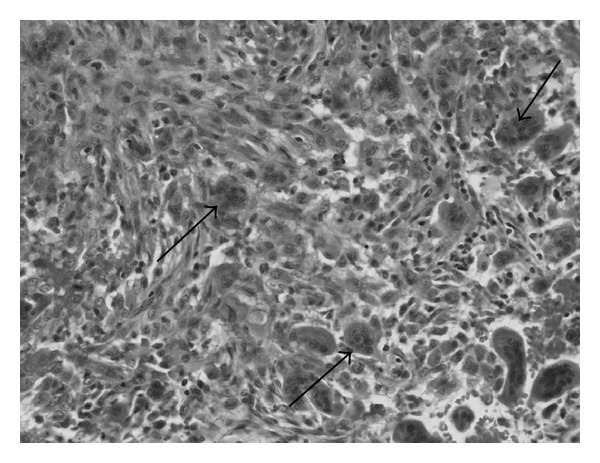
HE staining from the specimens of the tumor. Round and spindle-shaped mononuclear cells and numerous multinucleated giant cells. Arrow: giant cell.

Pathological examinations from specimens of the tumor revealed round and spindle-shaped mononuclear cells admixed with numerous multinucleated giant cells (Figure 4), characteristic of giant cell tumors.

## 3. Discussion

GCTs are derived from differentiated mesenchymal cells of the bone marrow [4], with 90% involving the epiphysis of long bones and less than 2% involving the skull [5–7]. Findlay et al. [8] reported 12 cases of giant cell tumors in the sphenoid bone, six in the petrous temporal bone, one in the middle fossa, and four elsewhere within the cranial vault. GCTs of the temporal bone are relatively rare.

There are no clinical symptoms specific to GCT. Symptoms can include local sharp pain, swelling, and exercise limitation of the joint resulting from a small bone fractures caused by the reduced bone strength. The main symptoms of giant cell tumors in the temporal bone are pain behind the ear, hearing loss, aural fullness, and facial weakness [8]. The tumors typically occur in the third to fourth decade of life, and recur at a high rate (10–20%) [9]. A very small proportion of patients with GCT (about 1%) may develop pulmonary metastases in addition to local bone destruction [3], and, moreover, in rare instances, a GCT may transform into a sarcoma [10].

On temporal bone CT, the giant cell tumors are typically seen as soft tissue specifications with osteolytic lesions causing bone erosion and sharp margins. MRIs of such tumors demonstrate isosignal intensity on T1-weighted images and low signal intensity on T2-weighted images and diffusion-weighted images, with the mass showing heterogeneous enhancement after intravenous gadolinium [11].

Histologically, giant cell tumors consist of three cell types, osteoclast-like multinucleated giant cells, round mononuclear cells, and spindle-shaped, fibroblast-like mononuclear cells [12]. In the present case, numerous giant cells like osteoclasts were seen histologically, and it is thought that the tumor was mainly derived from stroma cells scattered among the giant cells. However, it is difficult to identify the origin of tumor cells.

For orthopedists expert at recognizing bone and soft tissue tumors, the diagnosis of GCT is relatively easily based on the clinical symptoms, age, and CT and MRI images. However, since head and neck surgeons experience few cases of GCT, it may be difficult to diagnose when it occurs in the temporal bone. One must include giant cell tumor as a possible diagnosis when presented with enlarging temporal bone lesions.

Surgery is the only effective treatment for the GCTs of the temporal bone [13]. Broad excision is desirable to lower the recurrence rate, but with GCT originating from the temporal bone, it is often difficult to perform complete and radical extirpation. Despite the locally aggressive, highly recurrent nature [9], GCTs are benign tumors with a good prognosis after wide radical resection. There is not a well-defined accept protocol for treatment of GCT with chemotherapy, and the benefit of the chemotherapy was unproven [14]. The use of postoperative radiation therapy for incompletely resected tumors is controversial [15].

 We successfully extirpated the GCT using a transmastoid and middle fossa approach. The facial palsy had almost completely recovered 12 months later, and the hearing level was restored to normal. The patient is free of recurrence after 4 years, but long-term observation is still required.
